# Giving as repaying: towards an embodied ethics of living donor liver transplantation

**DOI:** 10.1007/s11019-025-10275-6

**Published:** 2025-06-05

**Authors:** Ya-Ping Lin, Huei-Ya Chen

**Affiliations:** 1 Department of Medical Humanities and Education, School of Medicine, National Yang Ming Chiao Tung University, Taipei City, Taiwan; 2 Institute of Public Health, National Yang Ming Chiao Tung University, Taipei City, Taiwan; 3 Department of Philosophy, National Chengchi University, Taipei City, Taiwan

**Keywords:** Living donor liver transplantation, Organ donation, Family, Embodied phenomenology, Narrative analysis, Ethics

## Abstract

This article explores the lived experiences and ethical complexities of the decision- and meaning-making journey involved in living donor liver transplantation (LDLT) through a case study of a young adult who donated part of his liver to his father. Utilising embodied phenomenology and narrative analysis, we present an in-depth exploration of personal stories and interconnected narratives that reveal the intricate layers and nuances inherent in child-to-parent LDLT within the Taiwanese sociocultural milieu. This study examines the embodied, relational, temporal and normative dimensions through a dynamic, iterative process of careful reading and analysis, from which four plotlines emerged [(1) indebtedness, (2) thrownness, (3) struggle for selfhood and (4) family seniority] along with a postscript. The findings illuminate the complex interplay of body, self, family, intergenerational dynamics and sociocultural norms throughout the decision-making process. The analysis aims to lay the groundwork for a refined framework for understanding concepts such as giving, relationality, agency, temporality and normativity within bioethical discourses on organ transplantation. Furthermore, the study offers insights for healthcare professionals to develop culturally sensitive approaches in LDLT care ethics and practice, with particular attention to vulnerability, relational autonomy and embodied intersubjectivity as normative foundations.

## Introduction

‘Besides morality, he is, after all, my father. I owe him for everything he’s given me since my birth’.

When asked about his decision to donate part of his liver to his father, 22-year-old college student Kuan-Ting explained that he felt a deep indebtedness to his father, which went beyond fulfilling a moral obligation and was rooted in their family bond. Kuan-Ting’s experience is not unique. According to Lin et al. (2021) and Lin (2023), the landscape of living donor liver transplantation (LDLT) in Taiwan is intertwined with complex family dynamics and broader sociocultural contexts. Given that Taiwanese law restricts living donor organ transplantation (LDOT) to family members within the fifth degree of kinship, it is a collaborative endeavour characterised by family-centred decision-making rather than donors’ or recipients’ individual choices. While LDOT is often morally justified on the grounds of the donor’s autonomy, recent research has contended that an understanding of autonomy that fails to consider the broader context and relationships does not adequately grasp the ethical implications of LDOT.

Existing research, mostly from the Western context, has primarily focused on parent-to-child living donation and has raised ethical concerns regarding decision-making autonomy within parent–child relationships (Filler et al. 2021; Forsberg et al. 2004; Freeman and Wightman 2018; Knibbe et al. 2007; Sarigol Ordin et al. 2017; Spital 2005; Ventura 2010; Zeiler et al. 2010). Scholars have identified a common experience faced by parents who are considering donating organs to their critically ill children, known as the ‘no-choice’ situation. Ethical frameworks have been developed to analyse this dynamic and explore the motivations and decision-making processes of donors who, driven by familial affections and obligations, perceive their decisions as necessary. The lack of choice is then explained and morally justified by reconstructing notions such as coercion, autonomy and agency (Biller-Andorno et al. 2001; Biller-Andorno 2011; Burnell et al. 2015; Crouch and Elliott 1999; Crowley-Matoka et al. 2004; Forberg et al. 2004; Fujita et al. 2004; Gordon 2011; Knibbe et al. 2007; Knibbe and Verkerk 2010; Otte et al. 2004; Spital 2001; Valapour 2008; Zeiler et al. 2010). For instance, Biller-Andorno (2011) argues that understanding voluntary donation merely as the absence of coercion fails to encompass the full spectrum of nonvoluntary decisions, particularly in living-related donation. In suggesting a relational model of the autonomous donor, Biller-Andorno also emphasises the need to be aware of potential ethical issues, such as the pitfalls associated with the ‘gift’ metaphor, including various family pressures and expectations that might compel sacrifices. While Fournier et al. (2013) seek to reexamine the role of ethical committees in LDLT, they correctly highlight the likely absence of free and informed donor consent in familial organ donation. Donors decide to donate before they receive information about associated risks. The decision appears to be an automatic response or leap, made in the moments following the announcement of the possibility of LDLT (Crowley-Matoka et al. 2004; McGregor et al. 2010). We share this perspective and stress the importance of addressing donors’ ethical uncertainties and concerns rather than merely assessing their decisional capacity and freedom of choice.

This article focuses on LDLT, as it serves as a ‘desperate remedy’ given that the patient’s life is at stake if a donor cannot be found within a short timeframe. Potential donors may experience heightened pressure in this life-or-death situation (Fujita et al. 2006; Moore 1989; Spital and Spital 1990). In contrast to studies that centre on parent-to-child organ donation, we specifically explore the experiences of adult children who donate organs to their parents, which reveal a dynamic that involves tensions and emotional struggles, as demonstrated in Kuan-Ting’s case. In many East Asian cultures, adult children commonly feel constrained by traditional expectations that are deeply rooted in family bonds and sociocultural norms. Furthermore, the intergenerational power dynamics prevalent in Taiwan profoundly influence children’s choices. Similar circumstances are observed in countries such as Japan and Korea (Bang et al. 2021; Fujita et al. 2006; Kusakabe et al. 2008; Nakamura 2007). Overall, research on child-to-parent organ donation in East Asia remains limited. This highlights the need for further investigation into the unique cultural, familial, and ethical challenges inherent in such cases.

This article presents a case study of a young adult who donated part of his liver to his father. The study was conducted using narrative analysis and embodied phenomenology. Unlike previous studies that focus primarily on donor autonomy or the corporeal and emotional connection between parents and children, our in-depth exploration delves further into the hidden intricacies and dynamics framed within the concept of family. We examine aspects such as body, self, relationships, power dynamics, choices and responsibilities as well as their relevance to the organ transplantation process. The goal is to lay the groundwork for a more thorough and comprehensive framework for understanding the lived experiences and specific ethical complexities of the decision- and meaning-making journey involved in child-to-parent LDLT within the Taiwanese sociocultural milieu.

## Methodology: embodied phenomenology and narrative analysis

### Theoretical perspective

Our case study is theoretically grounded in both embodied phenomenology and narrative analysis. This approach enables a comprehensive understanding of individuals’ lived experiences and meaning-making with respect to their bodies, perceptions, sense of self and life narratives within their social context. Husserl (1989) distinguishes between two aspects of the body: *Körper* (the physical body as an objective, material and natural entity) and *Leib* (the lived body as a subjective, experiential and personal aspect). According to Husserl, the lived body is an embodiment of subjectivity, consciousness and lived experience, and we engage with the world and experience and make sense of our surroundings through it. Merleau-Ponty developed a phenomenology of the body that argues for a non-dualistic account of human existence. Drawing on Husserl’s distinction, Merleau-Ponty uses the term ‘one’s own body’ (*le corps propre*), which enables a shared wholeness with others, underscores the mutual interrelation of human actions and emotions at the bodily level and articulates his conception of subjectivity as inherently embodied. The notion of embodied ‘being-in-the-world’ (*être au monde*) signifies that human beings are always engaged in relations with others and objects. As embodied subjects, we do not exist in isolation, facing the world alone, but rather experience ourselves in relation to others through bodily interactions (Merleau-Ponty 2012).

This study delves into the lived experiences of a young adult who has donated part of his liver to his father. These experiences encompass not only personal interactions but also the complex interplay of emotions, family dynamics, moral responsibility and sociocultural contexts. According to Mattingly and Garro (2000), ‘narrative analysis’, a method of understanding human experience through stories, offers a powerful means to comprehend human experiences constructed by personal and cultural resources. By telling one’s story, individuals provide a structured form for their lived experiences, enabling potential understanding (Frank 2012). It allows us to notice differences and diversity in people’s behaviour while considering temporal contexts and complex interactions (Polkinghorne 1995). The narrative analysis undertaken here seeks more than a mere thematic categorisation of individual experiences; rather, it revisits the empirical data collected through interviews and organises it into a concrete structure (Todorova 2007). In particular, the narrative includes diverse events, occurrences and actions of human life in an oriented progression. Within the narrative structure, these events are seamlessly integrated into a coherent chronological whole. Through processes such as storying and restorying, these events are woven into a clear storyline with a central theme, known as the plot. The plot unfolds through the intricate interplay of time sequences, human motivations, unforeseen events and evolving interpersonal and environmental contexts. This analysis aims to create understandable and cohesive stories (Polkinghorne 1995).

This analysis is not simply a post-data collection step; it is intrinsic to the entire research process. Coffey and Atkinson (1996) point out that narrative analysis relies on principled decisions shaped by the researcher’s problem awareness and theoretical perspective. Through the perspective of embodied phenomenology, our focus is directed towards how one is embodied in one’s family life. By revisiting and immersing themself in the empirical data, the researcher distils new concepts from existing ones, initiating preliminary discussions and advancing theory through dialogue (Coffey and Atkinson 1996). The study’s narrative analysis does not aim to create universal elements for pinpointing core constructs related to the issue of parent–child organ donation but to provide a fresh perspective into an individual’s lived experience within the world. The study combines embodied phenomenology and narrative analysis to offer a novel lens to understand subjectivity as embodied and embedded within family dynamics and sociocultural contexts during decision-making processes.

### Study design

This article presents findings from a qualitative ethical study examining family dynamics in LDLT in Taiwan (Lin et al. 2021) that involved participant observations during family meetings, transplant team discussions and psychological evaluations. Discussions were also held with transplant surgeons and liver transplant nurse coordinators. Our original study design was to invite both the donor and the recipient for interviews; however, unfortunately, the father declined to participate at the outset. Kuan-Ting, the donor, was invited by the transplant coordinators to participate in the study and provided written consent prior to the observations and two interviews. The initial interview took place the day before the transplantation surgery in the meeting room. Due to the COVID-19 pandemic, the second interview took place via Skype, precluding face-to-face interaction and direct behavioural observations. The in-depth semi-structured interviews, following a narrative form, were conducted to explore Kuan-Ting’s relationships with his father, mother and other family members as well as to delve into his family dynamics, school life and experiences in donation decision-making and the transplant evaluation process.

The original research intention was to conduct individual interviews. However, Kuan-Ting and his mother preferred joint interviews, and the interaction naturally unfolded in this manner. Despite the potential impact of her presence on Kuan-Ting’s narration, the researchers considered that, in the current context, her companionship allowed him to feel more comfortable and at ease during the interview process. This comfort stemmed not only from his mother frequently accompanying him to the hospital but also from their close relationship, which could bring forth rich insights during the interview. Therefore, the researchers concluded that her presence would not impede the analysis but instead contribute to Kuan-Ting’s narrative.

We enhanced our observations with reflective field notes taken by the first author that captured Kuan-Ting’s mother’s narratives and their interactions during the interview process. This approach aimed to provide a more multifaceted understanding of the themes embedded in Kuan-Ting’s narrative. It allowed us to delve deeper into the dynamics within the family context and gain a nuanced understanding of the implicit elements and unspoken aspects contributing to the family’s perspective on organ donation. Following Frank’s (2012) emphasis on embodied fieldwork as crucial for understanding narratives, the approach requires narrative analysts to immerse themselves fully in the bodily experience of the subject. Immediately after the interviews, the first author wrote reflective notes to capture the embodied impressions from the dialogue, and they helped us listen to Kuan-Ting’s story and understand his unique life situation within his family relationships. We read the whole transcript several times to analyse his narrative and then identified key events, conflicts and settings. Through iterative discussions, the plot was further organised in a chronological or logical sequence. Finally, we sorted out four plotlines to present a coherent and meaningful narrative structure.

## Embodying the self in relation: narratives of Child-to-Parent LDLT

### Kuan–Ting’s story

Kuan-Ting, a 22-year-old college student, grew up in a family of five in Chiayi, a city in southern Taiwan. His mother is a homemaker, and his father runs a family business and has a distant relationship with his family. Kuan-Ting has an easy-going yet cheerful personality. His relationship with his mother and two sisters is much like that of friends who openly discuss things with each other; however, he has little emotional interaction with his father. Upon entering university, Kuan-Ting moved to Kaohsiung to live independently. He sometimes returns to Chiayi during school breaks.

When Kuan-Ting’s father suddenly fell ill with acute liver failure and asked him to donate a part of his liver, Kuan-Ting was facing a challenging situation, as he was about to enter his final year of college and arrange his professional internship.

According to Taiwanese law, individuals aged 18 and over are eligible to donate part of their liver to relatives within five degrees of kinship. Although both of Kuan-Ting’s sisters met this criterion, concerns about their physical strength and the risk of scarring, given societal expectations regarding females, led the family to decide that Kuan-Ting should be the donor. Moreover, Kuan-Ting himself felt that, as the son, he had a particular responsibility to assume this role.

While Kuan-Ting expressed his willingness to donate to save his father’s life, he hoped to schedule the surgery to avoid interfering with his school exams and previously arranged internship schedule and affecting his classmates who were part of his internship group. However, due to the lack of direct communication between father and son, Kuan-Ting felt disrespected and experienced immense pressure, particularly from his elderly family members. Concerned about the potential stigma of being deemed unfilial towards his father, Kuan-Ting felt trapped between his desire for fair treatment and the pressure to conform to social norms of reciprocity and filial piety. Although he agreed to the donation, it was a decision he felt compelled to make. ‘Having no control of one’s own body’, or *shēn bù yóu jǐ* 身不由己, is a common Chinese proverb to metaphorically describe people who are predisposed to act in a certain way because of the social context in which they are embedded. The proverb was realised in Kuan-Ting’s story, as he found himself predisposed to donating an organ to his father, given the profound blood tie between them.

### Plotlines and postscript

Following a dynamic, iterative process of careful reading and analysis and extensive discussion between the authors, four plotlines emerged from the first interview: (1) indebtedness, (2) thrownness, (3) struggle for selfhood and (4) family seniority. Due to the limited opportunities for face-to-face interaction in the online interview, coupled with Kuan-Ting’s hazy memories a year after his surgery, his second interview narratives were less extensive than those from the first. The content of the second interview lacked richness in two key aspects: first, in terms of the chronological sequence, content and expression style, they could not be well integrated into the four plotlines to form a coherent story; second, an independent plot could not be developed from the second interview. Nevertheless, our examination of the interview revealed a discernible change in Kuan-Ting’s family relations and his self-understanding after the surgery. Therefore, we presented a postscript as supplementary data to enrich the analysis results.

#### Indebtedness: ‘After all, he gave me this body. So I have to repay him’

When Kuan-Ting learned that his father required a liver transplant, he initially faced uncertainties about his father’s health condition and, to some extent, blamed it on his father’s smoking and drinking habits. Yet, when asked about his reasons for donating, he replied with naturalness: ‘He is my dad, and I owe him for everything he’s given me since my birth’.

At first, Kuan-Ting was reluctant to frame his decision in terms of ‘morality’ or ‘filial piety’. Nevertheless, prompted by his mother’s suggestion, he expressed, ‘After all, he gave me this body, so I have to repay him’. This statement reflects his decision as rooted in a deep sense of gratitude and responsibility towards his father. Notably, Kuan-Ting described his relationship with his father as distant, likening him to a ‘nodding acquaintance.’ He elaborated on their interactions, providing further insight into their dynamic:

My relationship with my father is just okay, quite ordinary. It’s just like that, a nod or something, that kind of relationship. Although he didn’t express concern for me often, he did it occasionally. For example, if I hadn’t returned to Chiayi from Kaohsiung for a long time, he would ask the family when I planned to come back or leave and so on and so forth.

Based on Kuan-Ting’s response, his relationship with his father appears functional and stable. Despite lacking deep engagement and emotional expressiveness, it maintains a fundamental familial connection without significant conflict or heightened emotional intensity. He acknowledged its ordinary nature, describing it as an ‘okay relationship’ without profound affection. In addition, while Kaun-Ting, his mother and his sisters often openly discuss things with each other like friends, he noted that ‘there’s a gap’ between him and his father. Kuan-Ting recounted that he, his mother and sisters ‘live in one country’, while his older father ‘lives in his own country’. Especially since his father is not talkative and has a more macho personality, there is less communication between father and son. For example, instead of directly discussing his illness or needs with Kuan-Ting, his father repeatedly directed such inquiries to Kuan-Ting’s mother, asking, ‘Is he coming back to Chiayi?’

Although not close in terms of their interactions and emotions, Kuan-Ting believed that his ‘blood ties’ with his father were indissoluble and that this bond influenced his decisions. However, this initial instinctive response was called into question when both his father and uncle actively requested his donation without discussion. This led Kuan-Ting to shift from a sense of naturalness to one of reluctance; he even began to worry that if he did not donate, he would be perceived as unfilial. Furthermore, he started questioning the rationale behind the notion of ‘repaying his father for his body’ by donating part of his liver. The perplexity deepened when he recalled his grandmother saying that it made no sense to give someone your body and then request it back. This view added a layer of complexity to the decision, making it less straightforward and natural as Kuan-Ting navigated the medical process.

#### Thrownness: ‘All decisions were left to me and my family to coordinate’

As his father’s illness worsened, Kuan-Ting’s initial sense of responsibility shifted to confusion and helplessness. He found himself feeling thrown into a situation that disrupted the trajectory of his life, and he realised that he could not approach his father and the decision in the way he had initially planned.

To determine his eligibility as a donor and assess the compatibility of his liver with his father’s, Kuan-Ting underwent a series of medical examinations and discussions with his family and the medical team. The LDLT procedure in medical centres typically involves a series of evaluations for potential donors, including medical tests, a risk–benefit assessment, psychological and social evaluations, as well as ethical evaluations. A transplant coordinator is responsible for coordinating these evaluations and acts as the main point of contact between the donor, recipient, key family members and medical team throughout this process.

During the organ matching process, the coordinator assured Kuan-Ting that he would be informed of the official examination report and the result privately. However, before the report was released, his uncle and other family members became eager to know the result. Encountered with sudden and intense questioning from his family, Kuan-Ting found himself lacking the time and emotional space to reflect on his decision. Instead, he was under immense pressure and faced demands from multiple family members.

To respect the autonomy of both the donor and the family, the coordinator refrained from actively intervening or probing into the progression of their decisions and provided space for Kuan-Ting to think over his choice. However, as Kuan-Ting engaged in the intricate dynamics of discussions with family members, fielding their inquiries and participating in medical processes and conversations with the coordinator on his own, he found himself involved in extensive conversations with multiple parties alone. Kuan-Ting felt as if the medical team had simply ‘left all decisions’ to him and his family, with no one providing guidance.

Moreover, having a healthy body made him even less powerful in this situation. He recalled, ‘At that time, the MRI was completed first, and they said that if there were no significant issues, everything should be fine; there is no problem’. Despite medical professionals’ attempts to ease his worries, their ‘no problem’ stance left him adrift in conversations with his father and deepened his sense of helplessness. The role of a healthy son further ensnared him with the weight of familial expectations. These intertwined facts—he is a son, a junior family member and in good physical health—constituted the unique and intricate circumstances in which he was immersed, with no easy escape.

Despite ultimately choosing to be a donor, Kuan-Ting insisted on setting the timeline for the surgery. His father and other family members, however, pressed for an earlier date. He initially resisted, citing the doctor’s advice that the surgery was not urgent, but eventually yielded to their persistent requests. Reflecting on this process, Kuan-Ting admitted, ‘Well… my current feeling is… chaotic. It’s like everything came too quickly—just very sudden. It’s like my arrangement of time has been disrupted.’ As he navigated these discussions with his family, the rapid pace of events disrupted his sense of time and left him feeling disoriented.

#### Struggle for selfhood: ‘I have probably missed about three exams already’

Kuan-Ting’s distress stemmed not only from familial pressure but also from the neglect of his needs as a college student. Like any third-year college student, Kuan-Ting was expected to perform well academically, participate in social activities, secure internships and build a network for his future career. However, Kuan-Ting also had to juggle these demands alongside the complexities of being an organ donor.

The donation coordinator initially offered some flexibility—for example, Kuan-Ting considered postponing the procedure to the semester break. However, his father insisted on scheduling the surgery during the semester, resulting in him missing important tests. Opting to finish his final exams first, Kuan-Ting was also concerned about his grades, stating, ‘I’ve missed about three tests, the ones that normally count as regular grades’. Despite his efforts and considerations, his father did not seem to acknowledge the challenges Kuan-Ting faced in balancing his roles as his father’s donor and a college student.

Even though Kuan-Ting’s father and uncle claimed that the decision was his, they simultaneously pushed him to agree to the surgery. ‘Are you going to do it? Are you planning to do it?’ They bombarded him with the same questions every few days, ignoring his attempts to convey his uncertainty and emphasise the potential need to reschedule due to other commitments. Reflecting on the situation, Kuan-Ting voiced his frustration, ‘Shouldn’t I be the one to make the decision?’ He continued as follows:

But he kept saying, ‘It’s okay, it’s okay, you think about it, you think about it’. At that time, I felt… Both Dad and Grandpa would inquire if I had taken leave and for how long. I wanted to say, ‘But the leave period hasn’t arrived yet, and I still have some things to think about’. That’s why I kept getting asked if I’d taken leave or not. I felt that they had no respect for… I was at a loss as to what to do with my time. They gave me the idea that I had to take this time off, at least until he was released from the hospital.

The relentless questioning became overwhelming, gradually shrinking Kuan-Ting’s space to think, judge, and act independently of his father and other senior family members. Despite assurances that he could miss tests or take leave without any consequences, he found that others were determining his surgery schedule for him. This loss of control extended beyond just the timing of the surgery; it also left him feeling disregarded as a college student trying to balance academic and social responsibilities, ultimately leading to feelings of disrespect. In his own words, Kuan-Ting began to view his father’s and uncle’s insistent demands as a form of ‘invisible coercion’.

#### Family seniority: ‘He is my elder, and I dare not disrespect him’

Kuan-Ting’s vulnerability brings to the forefront the power dynamics between the younger and older generations, especially the authority held by the latter. He narrated that his family had an authoritarian interaction style, with his father and uncle often using a commanding approach to give instructions to their children. When his father required a liver transplant from someone within the family, his father and uncle directly demanded his donation, with no prior discussion or negotiation.

Faced with this patriarchal interaction and decision-making process, Kuan-Ting frequently found himself passively accepting it. He recalled a recent incident at the hospital where his uncle took pictures of him without his consent while he was having his blood drawn. He did not understand why his uncle acted this way, and it made him very uncomfortable, but he hesitated to express his feelings: ‘Because, after all, he’s my elder and I dare not, I dare not disrespect him’. During a one-on-one meeting with a social worker, he expressed his hesitation to donate at one point. However, he did not explicitly admit that it was due to pressure from his uncle. He explained it as follows:

Later on, we discussed matters related to liver donation as well as post-surgery affairs, and I was constantly troubled by thoughts about internships… Yeah and I started feeling unhappy. So, I asked the social worker to take them out and interview me separately… At that time, the social worker asked me why I originally didn’t want to donate. I told them, and later, they asked how I felt then. I said, ‘I just… I just have no choice.’ … I did tell them, I did tell the social worker. Actually, at first, I didn’t really want to donate… I was really, really angry at that time. *But I didn’t want to admit it was because of my uncle.* (emphasis added in italics)

Moreover, it was not only the elders’ decisions that made Kuan-Ting uncomfortable; his uncle’s incessant urging and unwarranted interference in his affairs were also irritating. Kuan-Ting emotionally expressed, ‘In normal times, he is basically not part of my life. To put it bluntly, he interferes in matters that have nothing to do with him’. Kuan-Ting’s mother echoed this sentiment, pointing out that despite being family, they do not spend much time together and only meet a few times a year.

Kuan-Ting was worried about his uncle’s opinion because he felt pressured to meet cultural expectations:

I’m also afraid of what others might say, like, ‘How can you act this way? He’s your father!’ …I’m afraid of being criticised for not being properly raised, as if my family didn’t teach me well.

Living in a culture that highly values respect and honour towards elders, Kuan-Ting feared being judged or criticised if he did not meet these expectations in his interactions with his father and uncle. In this situation, feeling the weight of sociocultural norms, Kuan-Ting chose to donate his organ.

#### Postscript: ‘Forget it! He’s my family anyway!’

A year after the surgery, Kuan-Ting reflected in the second interview that he had not noticed any significant differences in his life. The physical changes were minor, and earlier worries about grades, internships, and credits seemed to have faded. Regarding the decision to donate his organ, Kuan-Ting revealed a complex mix of emotions. While he felt he could donate, he admitted, ‘I felt I had to do it, but then it didn’t feel comfortable. I just didn’t really want to deal with it’. He recalled that many extended family members, aside from his immediate family, simply said, ‘Don’t think too much! Just go ahead and donate’, which only increased his sense of pressure.

Kuan-Ting felt even more uncomfortable because these family members ‘have a body to do the same thing’ as he did. In other words, they were all eligible to be donors as well. However, they acted as if it was none of their business. When these seniors actively offered him assistance after the surgery, such as a drive to the hospital, he politely declined. He explained that such help was unnecessary then, and he did not want to continue discussing donations with them during the drive.

In the day-to-day dynamics of his family life, explicit conversations about Kuan-Ting’s organ donation were rare. However, there was an underlying sense of change in his father’s demeanour and the overall family atmosphere. Kuan-Ting noticed that his father had become kinder after the surgery and was actively engaged with his children, which made Kuan-Ting feel ‘a little weird’. Kuan-Ting recalled, ‘He would actively come to me and try to talk to me, but I don’t think there was anything I could discuss with him’.

Having lived through this incident and its various unpleasant aspects, Kuan-Ting has made it clear that he no longer wishes to discuss it with others. As Kuan-Ting put it, ‘The past is the past’. He regards the matter as quite commonplace, simply involving repaying his family for the body they gave him.

It’s not a big deal; it’s nothing. I think it’s very ordinary. It’s just a matter of…* repaying a debt*?… Because at the beginning, I felt… my body was given to me by my family! (emphasis added in italics)

In addition, he explicitly expressed that bringing up the issue repeatedly only adds unnecessary pressure:

Since this incident, it’s been brought up for discussion, and I find it very stressful because I don’t want to talk about this kind of thing! It’s simply something that’s over and done with. Unless I bring it up myself, I don’t want to have it talked about again because *it’s just between me and my family*! There’s no need… there’s no need for everybody to bring it up. (emphasis added in italics)

Kuan-Ting also mentioned that during the transplantation evaluation process, it is acceptable for individuals other than family members, such as social workers, to engage in conversations about it. However, he emphasized that these discussions should not solely revolve around weighing the pros and cons of organ donation.

I believe it’s alright to discuss this topic, but please don’t make me feel… um, how can I put it… maybe… it’s fine to talk, but let’s not only focus on the benefits or drawbacks of donating or not donating. We can also have more casual conversations.

How does Kuan-Ting now interpret his donation decision and understand his relationship with his father? In the postoperative interview, when Kuan-Ting mentioned the metaphor of ‘repaying a debt’ again, his tone was noticeably more relaxed, and he even responded to the interviewer with a touch of humour.

Kuan-Ting: My family initially said it might be more like repaying a debt.

Q: May I ask who said that?

Kuan-Ting: Hehe, just another person who gave me the body.

Q: Oh?

Kuan-Ting: Yes!

Q: Another person who gave you the body?

Kuan-Ting: My mom! 〔Clearly intensifying the tone.〕.

Q: Oh! Haha, so she was advising you to think like that? Is that it?

Kuan-Ting: Advising me? No! She has always been telling me ‘It’s up to you! It’s up to you! It’s your call!’ She just said, ‘If you really don’t think you can handle it, then think of it as a form of repaying a debt’.

Q: Oh, I see. So, do you accept that perspective?

Kuan-Ting: Well, it’s okay! It’s fine.

In Kuan-Ting’s narrative, we find an intriguing juxtaposition. When asked what advice he would offer to someone facing a similar situation, he underscored the importance of thinking for oneself and honouring one’s own wishes.

I think that if you, as a child, want to donate, you have to be responsible for yourself because it’s your body! If you truly don’t want to, then don’t do it; just think for yourself and don’t worry about what other relatives or close family members say!

However, as he reflected on his own decision, Kuan-Ting’s response revealed a sentiment somewhat inconsistent with his earlier advice. Despite his initial reluctance, he ultimately decided to proceed with the donation, driven by a sense of familial duty.

Q: It seems like you were reluctant to do this. Why did you do it in the end?

Kuan-Ting: Eventually, I just thought, ‘Forget it! He’s my family anyway!’

Overall, Kuan-Ting’s lived experiences embodied a transition from feeling that he had no control over his own body to striving to make his own choices. He continues to practice taking responsibility for his body, navigating family dynamics and asserting his identity within his relationships. Upon reflecting on the meaning of organ donation, he not only gained an understanding of the depth of familial bonds and responsibilities but also started caring for his own body. While recognising that his body is inherently his own, he now sees the tangible connection it holds to his family. Essentially, Kuan-Ting’s journey epitomises a profound exploration of self, family and relationships, fuelled by the transformative experience of organ donation.

## Discussion

This study explored the multifaceted lived experiences of the decision- and meaning-making journey of a young adult who has donated part of his liver to his father. Utilising embodied phenomenology and narrative analysis, this study is the first of its kind, with the analysis offering unique insights into the profound interplay of selves, others and lifeworlds within the LDLT landscape in Taiwan. Our methodological, theoretical and empirical novelty lies in presenting a structured exploration of personal stories and interconnected narratives that reveals the intricate layers and nuances inherent in the lived experiences of child-to-parent organ donation within the broader sociocultural dynamic, examining the embodied, temporal and normative dimensions. The phenomenological inquiry involves understanding selfhood, subjectivity, temporality and freedom as embodied and situated in relations with others and the world, and the narrative analysis sheds light on the impact of family relationships on Kuan-Ting’s choices, internal struggles and negotiation of conflicting demands and values. By immersing ourselves in Kuan-Ting’s narratives, we gain deeper insights into the ethical complexities inherent in intrafamilial LDLT.

### The many faces of child-to-parent LDLT in Taiwan

This study’s findings reveal the multifaceted nature of child-to-parent LDLT in Taiwan, calling for a nuanced understanding of giving, relationality, agency, temporality and normativity within bioethical discourses on organ transplantation. These insights further underscore the need for culturally sensitive approaches in LDLT care ethics and practice.

#### Giving as repaying (*huán* 還)

Zeiler (2014a, b) critically examined four frameworks for understanding bodily exchanges: property rights, heroic gift-giving, sacrifice and gift-giving as aporia, which were developed from two cultural scripts that have dominated the discussion on organ donation (i.e. property rights and gift-giving). She argued that all these frameworks face difficulties in comprehending reciprocity and relational interdependence in the context of organ donation. Drawing on Merleau-Ponty’s phenomenology, Zeiler proposed the ‘intercorporeal framework of giving-through-sharing’ as a compelling alternative, which takes seriously the relational dimensions of human existence and co-existence, as well as the interconnectedness with others. This framework calls for a rethinking of the relation between self and other, highlighting the role of embodiment in ethical action and interaction. In a later study, Zeiler (2018) further proposed the concept of ‘bodily style of *being-together*’ to understand the complexities of parental live kidney donation. She argued that the co-inhabiting parents and children with end-stage renal disease have constituted a sedimentation of shared experiences that developed a unique bodily style of *being-together*. However, we argue that, while Zeiler’s account has the virtue of mapping onto our narrative analysis, it fails to adequately capture the primordial parent–child bond forged through blood ties and the asymmetry in intergenerational family dynamics. Kuan-Ting’s experiences unfold with a different relational perspective in LDLT: the naturalness of his donation is primarily rooted in the blood relation rather than emotional closeness to his father.

Our findings revealed that Kuan-Ting’s reasons for donation were deeply intertwined with his understanding of family ties, intergenerational reciprocity and social norms. A recurring narrative theme that emerged throughout our interviews was particularly striking: ‘My father gave me this body, and I have to repay (*huán *還) him’. As noted earlier, Kuan-Ting felt more obligated by the moral imperative stemming from blood kinship than familial affections. According to Confucian teaching, one receives one’s life and body from one’s parents. The idea emphasises our parents’ contribution to our existence and implies a profound sense of gratitude for and dependence on them, underscoring the significance of respect and gratefulness, as well as the importance of repaying them for their care and nurturing (Pound 1954; Rosemont Jr. and Ames 2008; Sung 2003).

Within this framework, organs are not perceived as replaceable body parts but as ‘flesh and blood’ (*gǔ ròu* 骨肉) that constitute family and kinship. This insight also prompts us to reconsider the understanding of the body beyond concepts of ownership and property rights. In deciding to donate, Kuan-Ting was not only considering his own body but also gaining insight into himself as a relational being, shaped by the undeniable presence of his father and the indissoluble blood ties between them (‘After all, he is my father’). Grounded in this ontological blood relation, children are forever indebted to their parents. Kuan-Ting viewed the act of giving his body as a form of repayment within the parent–child relationship as natural and self-evident. It was his way of repaying his father for ‘bestowing his body and life’, which he considered ‘natural and right’ (Lin et al. 2021). Kuan-Ting’s decision was not simply an individual choice; rather, it reflected his deep-rooted relationality with his father and his commitment to upholding familial values of reciprocity. We call this ‘*giving as repaying* (*huán* 還)’ which stems from the belief that people are in perpetual debt to their parents who gave birth to them and raised them from infancy.

Furthermore, our study brings attention to an issue often overlooked in Western perspectives: the significant impact of intergenerational power imbalances on the choices and experiences of adult children. In East Asian culture, ‘seniority in the family’ (*bèi fen *輩分) obliges the younger generation to respect the older generation. Within this context, family dynamics often follow a specific framework that emphasises honouring and respecting elders, giving decision-making authority to the older generation and favouring indirect communication (Sung 2001). During a family meeting attended by Kuan-Ting and his father, uncle and mother, the first author observed that his father and uncle tended to dominate the conversation, while Kuan-Ting mainly remained silent, and his mother only spoke about matters concerning daily life and postsurgical care. These interactions at the scene mirrored the underlying dynamics within Kuan-Ting’s family.

As depicted in the plotlines, both the elders and society perceived child-to-parent organ donation as a way of fulfilling the moral duty to repay one’s parents. These cultural norms contributed to Kuan-Ting’s fear of potential judgment or criticism if he did not conform to the expectations in his interactions with his uncle and father. The hierarchical and asymmetric nature of parent–child relationships placed Kuan-Ting under the constraints of both the moral imperative of seniority and a sense of indebtedness, in contrast to the parenthood moral imperative for decision-making under the Western context of parental living kidney donation (Zeiler et al. 2010).

#### Ethical subject as embodied and socially embedded self

Our study revealed that the decision-making process surrounding child-to-parent LDLT involved complex family dynamics and diverse roles, which posed challenges for Kuan-Ting. His multiple personal identities and their societal expectations added layers of complexity to his understanding of self and place within the family. Our narrative analysis clearly showed that he struggled to reconcile his roles as a son who was expected to donate his liver to save his father and as a college student with academic and internship responsibilities. While his father and elders urged him to undergo surgery promptly, he also had to meet his academic commitments with limited time and resources. Additionally, because the family believed that the father’s life held greater importance than the son’s career planning, Kuan-Ting’s personal needs and circumstances were disregarded. Despite his efforts to balance his responsibilities, he felt constrained and powerless, seen only as a healthy son who should donate his liver, with no further consideration given. This imposed situation left Kuan-Ting feeling like he had no choice.

Some may question whether Kuan-Ting could have chosen not to donate, defying his father’s authority and refusing to be coerced by his family, thereby asserting his autonomy. However, the mainstream representation of an autonomous agent, which assumes that they are rational, independent, self-interested and transparent, with fully conscious personal wishes and volition, has been critiqued by relational theorists (Christman 2004; Crouch and Elliott 1999; Mackenzie 2014; Mackenzie and Stoljar 2000; Westlund 2009). Our findings align with the relational stance, particularly in highlighting that the individualistic picture of agency oversimplifies the difficulties experienced within relationships and often blames individuals for their perceived lack of rational competency or critical reflection. As illustrated by Kuan-Ting’s experience, the decision-making process is not a straightforward endeavour conducted by a singular, unified subject but rather a dynamic interplay of multiple identities that requires continuous navigation and negotiation, along with the associated responsibilities. Throughout the process, Kuan-Ting repeatedly expressed his willingness to compromise for his father while also desiring more respect from his family.

Furthermore, our study underscores the significance of the bodily dimension in understanding the relational self during the decision-making process. Scholars emphasise the importance of considering the non-cognitive dimensions of the autonomous agent, as these aspects cannot be adequately explained solely through reason and cognitive self-reflection in the medical decision-making process (Kall and Zeiler 2014; Lewis and Holm 2023; Mackenzie 2001). Taking an embodied phenomenological perspective, our bodies serve as the foundation for perceiving and engaging with the world, shaping our values and motivations even before we consciously recognise them. Thus, Kuan-Ting’s lived experience is not simply a conflict of external role obligations; it is also a manifestation of how his embodied self navigates and internalizes complex relational expectations. According to Crowell (2013), one’s practical identity cannot be simplified through reflective role-taking but is rather expressed through how one’s body intentionally responds to the environment in specific ways. The normative meaning of practical identity must be grasped by understanding the context of a person’s being in the world. Crowell also acknowledges that most people’s practical identities are complex and constantly evolving, encompassing multiple descriptions or roles. In this light, Kuan-Ting’s predicament reveals another facet of intrafamilial organ donation: it is not simply a matter of saving lives but also involves reevaluating one’s practical identities within a complex network of relations with oneself, others and the world.

#### Temporal experiences

Our study revealed that child-to-parent LDLT involves intricate temporal experiences, encompassing coping with a parent’s illness, making the decision to donate, undergoing medical procedures, and adapting to life changes. Brough (2001) notes that while objective time is fixed, subjective time can expand or contract, particularly in extraordinary circumstances such as severe illness. This distinction became especially pronounced for Kuan-Ting, who experienced intense time pressure throughout the donation process. As his father’s liver failure progressed rapidly, his elders emphasised the urgency of surgery, urging him to conform to the constraints of objective time. Despite his preference to postpone the procedure, their insistence disrupted his sense of time, underscoring the generational disparities in temporal perception within the family.

We found that younger and older generations operate on distinct temporal frameworks when navigating child-to-parent organ donation. For Kuan-Ting, the decision to donate an organ represented a significant disruption to his planned trajectory, requiring him to reorient his schedule and accommodate medical procedures and personal concerns within a compressed timeframe. His elders, however, downplayed his concerns, remarking, “I’ve been through it too; it’s nothing.” This dismissive attitude not only reflects an inherent power imbalance within the family hierarchy but also underscores the generational divergence in temporal experience.

Life course theory provides insight into these differences, recognising that individual experiences are shaped by anticipated biomedical events, personal circumstances, and societal norms (Exley and Letherby 2001). At this critical juncture in his early twenties, Kuan-Ting is transitioning from adolescence to adulthood, navigating identity formation, academic aspirations, career ambitions, and evolving interpersonal relationships. In contrast, his elders, having already established their careers, raised families, and accumulated life experience, perceive time from a markedly different standpoint.

As Adam (1990) questions the linear conception of time and highlights the multifaceted nature of temporal experiences, our study underscores the intricate interplay between subjective and objective time. This perspective invites a reconsideration of the ethics of LDLT, situating it within the evolving temporal fabric of family relationships. By framing decision-making within these layered temporal experiences, we gain a deeper understanding of the ethical complexities faced by donors like Kuan-Ting, where the intersection of medical urgency, personal life course, and intergenerational perceptions of time shapes a nuanced ethical landscape.

### Towards an embodied ethics of LDLT

Traditional debates on living organ donation have primarily focused on informed consent, risk–benefit assessment and voluntary choice within a principle-based ethical framework (de Villa et al. 2003; Singer et al. 1989; Singhvi et al. 2016; Walton-Moss et al. 2005; Wright 2004). However, by delving into the lived experience, we acknowledge that the challenges faced by donors such as Kuan-Ting cannot simply be reduced to conflicts between moral principles or ethical dilemmas. Instead, these experiences involve how individuals, as embodied selves, engage with the incident within the relational fabric of their lives, navigating multiple interpersonal dynamics and taking actions in response.

In this context, we draw on Weiss’s (1999) concept of embodied ethics to illuminate and comprehend the ethical implications of the lived experiences of intrafamilial LDLT. Weiss contends that embodiment entails intercorporeality, where the body is not a self-contained entity with fixed boundaries but rather interdependent, forming the foundation of individual embodiment through relationships. Based on our proposed idea of ‘*giving as repaying*’, we argue that the motive for child-to-parent organ donation extends beyond a mere recognition of filial piety as a Kantian categorical imperative. Instead, it stems from the bodily imperatives that emerge out of the intercorporeal and interdependent flesh-and-blood bonds between children and their parents.

Furthermore, we advocate for a shift in focus from addressing autonomous decision-making to the practices of family ethics in contemplating LDLT in its nuance. Family involves relationships, emotions, responsibilities and care. Kuan-Ting’s narratives highlight the crucial role of the primordial parent–child bond and the asymmetry in intergenerational dynamics. The creation and nourishment of life and body, the perpetual indebtedness in parent–child relations, the intercorporeality, interdependency and reciprocity within familial contexts, as well as the conflicts and balancing of multiple roles of adult children over time, constitute the core of our framework for examining the ethical implications of LDLT. Adult children’s decisions and actions regarding organ donation represent intercorporeal experiences intertwined with bodily and ethical relationships. The difficulty of deciding on organ donation arises not only from the act of donating but also from the challenges the embodied selves face amidst the many changes in life and the inherent familial and social responsibilities they cannot avoid.

### Revisiting the ‘No-choice’ theme: making sense of the predicament in family

A central ethical concern regarding living donation is the potential for coercion or involuntariness, which can compromise the donor’s autonomy. Several studies in Western contexts have reported that emotional connections and parental duties can create a perception of ‘no-choice’ situations where donation is seen as almost predetermined by the parent–child relationship rather than freely chosen (Zeiler 2014a). In the case of child-to-parent LDLT in Taiwan, familial bonds also hold significant cultural and ethical weight. However, the question of whether this ‘natural and right’ decision of donating one’s organ, which is often driven by the flesh-and-blood bonds, is truly autonomous becomes even more complex, even with a desire to help a parent in need.

Distinct from the discussions on the ‘no-choice’ theme in the existing literature, we contend that the situation in Taiwan, especially in the case of Kuan-Ting, presents a more nuanced perspective. The conceptualisation of ‘*giving as repaying*’ carries significant normative weight, framing child-to-parent organ donation as an obligation to reciprocate parents for granting life. However, this framing can also lead to coercive pressures on donors, as they may feel compelled to participate in donations due to familial obligations. As Kuan-Ting articulated, despite sometimes feeling emotionally distant from his father, the very fact that his parents gave him life underscored an inherent familial bond. For him, the shared bodily and existential connection, encapsulated in the concept of intercorporeality and interdependency in parent–child relationships, formed the bedrock of his decision to donate his organ.

The crux of Kuan-Ting’s decision lay in his predicament of being unable to refuse, or *shēn bù yóu jǐ* 身不由己: while recognising his obligation to repay his father and being willing to donate, he felt constrained by the overpowering pressure from his elders. This situation illustrates the complex nature of filial practices in East Asian cultures, where adult children’s responsibilities to their parents are intertwined with intergenerational hierarchy, further complicated by their conflicting roles. These multifaceted aspects of self constituted Kuan-Ting’s relational identities in the decision-making process, making it difficult for him to fully grasp the extent of his choices.

A more profound issue emerges regarding the dual nature of one’s body, both as one’s own and as part of one’s parents or family. A person’s body is their own—it is the core and medium through which they exist, perceive, experience, perform and build relationships. However, when a person’s body is simultaneously considered to belong to their parents, and they are expected to repay their parents in the context of child-to-parent LDLT, the immense urgency and moral pressure may make the person feel coerced in their choice. This feeling echoes Kuan-Ting’s grandmother’s comment that it makes no sense to give someone your body and then request it back. Such dynamics may result in emotional detachment, indifference after the event, and a reluctance to revisit the experience, as it reflects a fracture in one’s self-identity.

Ultimately, Kuan-Ting’s decision-making journey revolved around the idea of family, initially focusing on the blood ties between parent and child but extending beyond mere biological relatedness. It encompassed moral commitments, responsibilities, and Kuan-Ting’s interpretation and understanding of the situation (Lindemann et al. 2019). Despite facing compromises and challenges, Kuan-Ting valued his family and cared for his family members, including his sisters. He chose to prioritise his familial duties, concluding, ‘Forget it! He’s my family anyway’.

Therefore, the lack of choice in this case was not merely the absence of options under coercive conditions. From Kuan-Ting’s perspective, it was the obligation to reciprocate that he framed as meaningful in his decisions. This, to some extent, also explains why Kuan-Ting advised others facing similar circumstances to follow their own will, though he remained deeply bound by family ties. The situatedness of the interplay of the biological, affective, temporal, expressive and sociocultural horizons of Kuan-Ting’s journey weaves the fabric of meaning and bodily commitment that determine his position in the world. It draws into focus the embodied, situated and relational ways in which subjects engage with a shared world.

### Vulnerability, relational autonomy and embodied intersubjectivity as normative foundations of LDLT care

Crouch and Elliott (1999) raise a critical ethical problem of parent-to-child LDLT: ‘Should a transplant team offer a parent, under circumstances of uncertainty, the opportunity to donate part of her liver to her desperately ill child?’ (p. 276). Objectors argue that parents should not be offered this option because their emotional ties and moral obligations towards their child might make refusal extremely difficult. However, Crouch and Elliott contend that neither love nor conscience constrains parental autonomy; instead, these elements express their identity as family members. Similarly, Knibbe et al. (2007) argue against perceiving a lack of choice and emotions as compromises of voluntariness and suggest that these should be understood as signs of commitment. While both Crouch and Elliott and Knibbe et al. adopt a relational view of moral agency, the latter is more critical, emphasising the importance of evaluating the quality of family relations. Based on our findings regarding child-to-parent LDLT in Taiwan, we align with Knibbe et al. and further underscore the need for healthcare professionals to pay attention to family commitment and relations.

Taiwanese society is considered family-oriented, although the concept of family is evolving (Yi and Chang 2019). Many adult children may readily become organ donors to save their parents, driven by familial bonds and their sense of responsibility, akin to Kuan-Ting’s articulation of repaying a debt or acknowledging indebtedness. However, this willingness can sometimes obscure an underlying reluctance, as characterised by Ross and Thistlethwaite (2021) as ‘the deferential vulnerability’. This form of vulnerability is often self-imposed and less formal, suggesting that potential living donors may exhibit deferential behaviour patterns that mask their true feelings about being a donor. In families with unequal power dynamics, it is crucial to protect the vulnerable members from undue pressure or exploitation by influential family members like parents or elders. Throughout the process, Kuan-Ting experienced immense pressure from his elders and society; he felt obligated to donate once he was deemed a suitable donor, facing disrespect and oppression due to communication gaps with his father. Additionally, differing temporal perceptions between generations may lead to conflicts in understanding and, more importantly, potential harm. All these highlight the need for healthcare professionals to provide donors with more structured support and guidance, recognising the complex emotional and social pressures they face.

LDLT presents a unique situation for the healthcare team, requiring care for both donors and recipients with distinct medical and ethical considerations, as well as their interconnected needs and dynamics. Rather than focusing solely on the care of the recipient as a patient, Fournier et al. (2013) argue for the importance of providing follow-up care that addresses medical, psychological, and social aspects, thereby empowering donors in their recovery. Otte et al. (2004) emphasise that the emotional distress and postoperative pain experienced by donors should be treated as seriously as those of recipients. The healthcare team must also carefully avoid imposing moral pressure on potential donors during medical-patient communication. Knibbe et al. (2007) and Zeiler (2009) suggest that professionals should create space for donors to understand their emotions, helping them recognize the potential exploitation or domination in the donor–recipient relationship and to comprehend the meaning and value of their decision. However, these studies primarily address parent-to-child donation and offer suggestions for caring for parental donors. Considering the unique family dynamics in Taiwanese society, care for child-to-parent LDLT should address the multifaceted vulnerabilities of the adult child donor, including issues related to identity, relational dynamics, temporal experiences, and challenges associated with power imbalances.

In our case, the healthcare team detected the tension within Kuan-Ting’s family dynamics. Nevertheless, leaving the decision entirely to Kuan-Ting out of respect for him and his family made him feel helpless despite his willingness to donate. His helplessness reflects the fact that he felt thrown into a predicament where he had no space to understand his own actions, interpret his emotions and digest his family members’ thoughts and concerns. Based on our analysis, merely encouraging adult children to choose not to donate out of obligation or refraining from interfering in their decisions under the principle of objectivity and neutrality poses risks, especially in the context of East Asia. To enhance LDLT care, we share the recommendations proposed by Ross and Thistlethwaite (2021). That is, healthcare professionals should incorporate a relational approach to autonomy, going beyond the provision of neutral medical information and empowering potential living donors by fostering open and candid family discussions where their particular vulnerabilities can be adequately addressed.

In our view, Kuan-Ting’s vulnerability offers insight into the complexities of a person’s lifeworld, not just his deferential position within the family but also his multifaceted existential questions. These are not mere problems to be solved but lived experiences to be understood. Healthcare professionals should recognise donors as embodied subjects and be sensitive to their engagement in a shared world. As Weiss (1999) eloquently articulates, the moral significance lies not in detachment from others but in our relations with them. Relationships do not hinder autonomy but create the necessary conditions for genuine autonomy to emerge. Our findings reveal that the decision to donate an organ is not a one-off moral choice. Rather, it is an ongoing meaning-making journey that involves evolving family dynamics, continuous ethical deliberation and a redefinition of identity and relationships over time. We should approach the child-to-parent LDLT as a temporally lived situation and embodied intersubjective dynamics and address the complex ethical considerations it entails. It requires the logic of care to help donors, recipients and their families adjust to realistic limitations, engage in relational life and avoid oversimplifying the complexities of medical situations (Mol 2008). Overall, we propose transitioning from the ethical emphasis on individual choice towards a more attentive consideration of the intricacies of family dynamics while also being sensitive to the individual’s need to maintain their sense of self within the family. Healthcare team should recognise the familial dynamics and cultural nuances inherent in Taiwanese society, particularly the hierarchical structure and the importance of respecting elders. Besides medical care, emotional and psychological support should be integral throughout the transplantation journey. Additionally, conversations with donors should not revolve solely around the decision to donate. As Kuan-Ting suggested, focusing only on the donor’s willingness to donate might make them feel that the team is not genuinely concerned about their well-being, which can lead to discomfort. Understanding these lived experiences of donors, recipients and their family members enables healthcare professionals to offer enhanced support, ensuring their voices are truly heard and respected and promoting ethical and empathetic LDLT care.

## Conclusion

This study offers a nuanced exploration of the lived experiences and ethical complexities of child-to-parent LDLT through an embodied phenomenological and narrative analysis. By examining the case of Kuan-Ting, we reveal how self, family, intergenerational dynamics, embodied vulnerability, relational autonomy, and sociocultural norms intertwine to shape the decision- and meaning‐making process in intrafamilial LDLT within the Taiwanese context.

Our findings underscore that child-to-parent LDLT is not merely an individual choice but embedded in relational interdependence and familial obligations. The concept of ‘giving as repaying’ illustrates how intercorporeal bonds between parents and children, along with moral responsibilities, influence donor experiences, while also exposing the ethical tensions and vulnerabilities that donors face in navigating these expectations. Kaun-Ting’s case demonstrates that pressures from senior family members, conflicting personal commitments, and implicit moral expectations intersect, shaping a complex ethical landscape where the decision to donate is part of an evolving relational and temporal process that challenges conventional autonomy- and principle-based bioethical frameworks.

Building on these insights, we advocate for a relational and embodied ethics approach in LDLT care. By shifting the focus from individual autonomy and informed consent to the relational, embodied, and intersubjective dimensions of care, we can better capture the donor’s lived experiences. Healthcare professionals should foster open, supportive, and culturally sensitive dialogues that enable potential donors to articulate their concerns, clarify their relational commitments, and navigate the emotional burdens inherent in evolving family dynamics during the transplantation process. We call for further inquiry into the intersubjective and intercorporeal dimensions of LDLT, with the aim of refining ethical frameworks and promoting compassionate, context-sensitive practice. In addition, future studies might explore how these dynamics unfold across diverse sociocultural contexts and how they continue to shape the donor’s embodied subjectivity and ethical self-understanding over time.
